# Self‐Illuminating NIR‐II Chemiluminescence Nanosensor for In Vivo Tracking H_2_O_2_ Fluctuation

**DOI:** 10.1002/advs.202207651

**Published:** 2023-06-13

**Authors:** Shiyi Zhang, Hao Yuan, Shengchun Sun, Chunlian Qin, Qiming Qiu, Yuyan Feng, Yongjie Liu, Yang Li, Lizhou Xu, Yibin Ying, Ji Qi, Yixian Wang

**Affiliations:** ^1^ School of Biosystems Engineering and Food Science Zhejiang University Hangzhou 310058 China; ^2^ Key Laboratory of Intelligent Equipment and Robotics for Agriculture of Zhejiang Province Hangzhou 310058 China; ^3^ ZJU‐Hangzhou Global Scientific and Technological Innovation Center Hangzhou 311215 China; ^4^ Children's Hospital Zhejiang University School of Medicine National Clinical Research Center for Child Health National Children's Regional Medical Center Hangzhou 310052 China; ^5^ Frontiers Science Center for Cell Responses State Key Laboratory of Medicinal Chemical Biology Key Laboratory of Bioactive Materials Ministry of Education and College of Life Sciences Nankai University Tianjin 300071 China

**Keywords:** optical imaging, second near‐infrared chemiluminescence, self‐illuminating

## Abstract

Chemiluminescence (CL) imaging, as an excitation‐free technique, exhibits a markedly improved signal‐to‐noise ratio (SNR) owing to the absence of an excitation light source and autofluorescence interference. However, conventional chemiluminescence imaging generally focuses on the visible and first near‐infrared (NIR‐I) regions, which hinders high‐performance biological imaging due to strong tissue scattering and absorption. To address the issue, self‐luminescent NIR‐II CL nanoprobes with a second near‐infrared (NIR‐II) luminescence in the presence of hydrogen peroxide are rationally designed. A cascade energy transfer, including chemiluminescence resonance energy transfer (CRET) from the chemiluminescent substrate to NIR‐I organic molecules and Förster resonance energy transfer (FRET) from NIR‐I organic molecules to NIR‐II organic molecules, occurs in the nanoprobes, contributing to NIR‐II light with great efficiency and good tissue penetration depth. Based on excellent selectivity, high sensitivity to hydrogen peroxide, and long‐lasting luminescence performance, the NIR‐II CL nanoprobes are applied to detect inflammation in mice, showing a 7.4‐fold enhancement in SNR compared with that of fluorescence.

## Graphical abstract

A self‐luminescent nanoprobe designed by cascade energy transfer, including chemiluminescence resonance energy transfer and Förster resonance energy transfer, contributes to the second near‐infrared (NIR‐II) luminescence with great efficiency and good tissue penetration depth. The NIR‐II chemiluminescence nanoprobe offers an excellent signal‐to‐ratio with a 7.4‐fold enhancement compared with fluorescence for tracking H_2_O_2_ fluctuation in vivo.

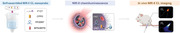

## 1 Introduction

Optical imaging has emerged as an influential technique for the real‐time monitoring of living physiological processes owing to its benefits of non‐ionizing radiation, high spatiotemporal resolution, minimal invasiveness, and fast feedback.^[^ ^1^ ^]^ Fluorescence imaging as a common modality of optical imaging, which requires an excitation light source to excite fluorescent probes, has been widely used. However, excitation light can induce a range of adverse effects, including tissue auto‐fluorescence,^[^ ^2^ ^]^ conversion of photons to heat derived from water adsorption,^[^ ^3^ ^]^ and inhomogeneous light illumination,^[^ ^4^ ^]^ contributing to compromised imaging quality or harmful to the living body. Chemiluminescence, an excitation‐free method in which an internal energy donor from chemical processes has been substituted for the excitation source, is gaining increasing traction as a solution to these problems. Various chemiluminescence systems based on luminol,^[^ ^5^ ^]^ lucigenin,^[^ ^6^ ^]^ and peroxyoxalate^[^ ^7^ ^]^ have been developed. Among these, the peroxyoxalate‐based chemiluminescence (POCL) system is widely used in biological diagnosis owing to its high chemiluminescence efficiency and excellent selectivity for hydrogen peroxide (H_2_O_2_) analysis. In a typical POCL system, a peroxyoxalate chemiluminescent substrate like bis[2,4,5‐trichloro‐6‐(pentyloxycarbonyl)phenyl] oxalate (CPPO) reacts with H_2_O_2_ to form a high‐energy intermediate (1,2‐dioxetanedione, DOD). Via the chemically initiated electron‐exchange luminescence (CIEEL) mechanism, the intermediate enables excite nearby fluorescent molecules to emit luminescence.^[^ ^8^ ^]^ Owing to the POCL luminescence mechanism, POCL systems frequently emit luminescence in the visible range and suffer from the problem of low penetration depth.^[^ ^9^ ^]^ To enhance the performance of the penetration, several attempts have been undertaken to extend the emission to longer wavelengths. The effective strategy is the integration of CRET and FRET mechanisms, which use acceptors to transfer the high energy generated by CRET emission systems to produce a red‐shifted signal. A pioneering study performed by Pu et al. utilized a sequential CRET‐FRET process between an energy substrate bis(2,4,6‐trichlorophenyl) oxalate (TCPO) and two fluorescent molecules poly[(9,9′‐dioctyl‐2,7‐divinylene‐fluorenylene)‐alt‐{2‐methoxy‐5‐(2‐ethylhexyloxy)‐1,4‐phenylene}] (PFPV) and silicon 2,3‐naphthalocyanine bis(trihexylsilyloxide) (NIR775).^[^ ^10^ ^]^ By integrating TCPO‐to‐PFPV CRET and PFPV‐to‐NIR775 FRET, the nanoprobes enabled the luminescence emission in the NIR‐I optical window (773 nm). To date, many POCL probes with NIR‐I emission have been designed and synthesized for a variety of in vivo imaging applications.^[^ ^11^ ^]^

The aforementioned probes, nevertheless, are unable to create detection territories in the NIR‐II range. Up to now, only a few publications on the successful development of chemiluminescence probes with NIR‐II emission have been reported. Fan et al. first reported the use of two fluorescent molecules including BTD540 and BBTD700 to integrate consecutive CRET and FRET to convert the high energy released from DOD into NIR‐II photons.^[^ ^12^ ^]^ Effective NIR‐II chemiluminescence depends on the wide Stokes shift region more than 100 nm as well as the extraordinarily high FRET efficiency between BTD540 and BBTD700. Research on chemiluminescence NIR‐II nanoprobes is still in its infancy, and their luminescence efficiency and duration time require further improvement. To construct a NIR‐II nanoprobe with high luminescence efficiency, fluorescent molecules used as energy acceptors and donors must meet fundamental requirements. First, the energy acceptors for CRET and FRET should have large absorption cross regions and a high quantum yield. Next, the large Stokes shift and extremely high FRET efficiency of both dyes enable the extension of the emission to NIR‐II wavelengths. Semiconducting polymers, as a new class of optical reagents, possess unique advantages, including large absorption coefficients, good energy match for DOD, excellent photostability, and good biocompatibility,^[^ ^13^ ^]^ making them ideal energy acceptors for CRET. Aggregation‐induced emission luminogens (AIEgens), another novel optical imaging probe, possess biocompatibility, photostability, and a particularly large Stokes shift compared with conventional fluorescent molecules,^[^ ^14^ ^]^ showing great potential for application as donor acceptors in CRET and donors in FRET.

Thus, in this study, by taking advantage of semiconducting polymers and AIEgens, we designed and synthesized a NIR‐II chemiluminescence nanosensor for tracking H_2_O_2_ fluctuation in vivo. The NIR‐II CL nanoprobes were prepared by nanoprecipitation of the chemiluminescent substrate CPPO, semiconducting polymer poly[2,7‐(9,9′‐dioctylfluorene)‐alt‐4,7‐bis(thiophen‐2‐yl)benzo2,1,3‐thiadiazole] (PFODBT), and AIEgens BPN‐BBTD into amphiphilic polymer pluronic F127. Owing to the high absorption efficiency of PFODBT, large Stokes shift and good energy level match between PFODBT and BPN‐BBTD, the NIR‐II CL nanoprobes enabled generate high‐efficiency chemiluminescence emission in the NIR‐II region. The nanoprobes showed improved tissue penetration depths when compared to that in the visible and NIR‐I regions. In addition, owing to the lack of excitation light, chemiluminescence avoids autofluorescence, overheating during long‐time imaging, and inhomogeneous light illumination. Based on excellent selectivity, high sensitivity to H_2_O_2_, and long‐lasting luminescence performance, the NIR‐II CL nanoprobes were applied to detect local inflammation in mice, showing a 7.4‐fold enhancement in SNR compared with that of fluorescence.

## 2 Results and Discussion

**Figure 1:**
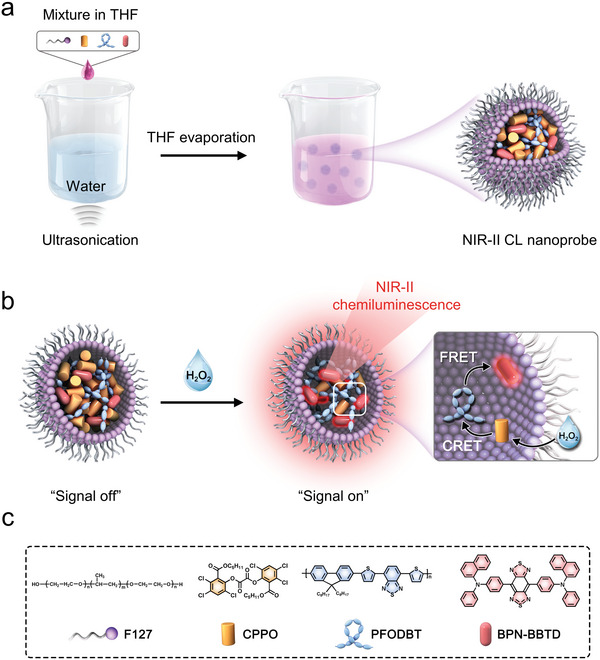
a) Schematic procedures for synthesizing NIR‐II CL nanoprobes. b) NIR‐II chemiluminescent mechanism of NIR‐II CL nanoprobes. c) Structures of F127, CPPO, PFODBT, and BPN‐BBTD used for synthesizing NIR‐II CL nanoprobes.

The NIR‐II CL nanoprobe was synthesized using a nanoprecipitation method (**Figure** **1**a,c). Three hydrophobic molecules, CPPO, PFODBT, and BPN‐BBTD, were embedded in the hydrophobic cavity of the amphiphilic triblock copolymer F127. The outer shell of F127 gives the nanoprobes good water solubility and biocompatibility, making them suitable for sensing in aqueous solutions. The three encapsulated sensing moieties in the nanoprobe generated NIR‐II chemiluminescence by combining two cascade energy‐transfer processes (CRET and FRET) (Figure 1b,c). CRET refers to the energy‐transfer process from CPPO to PFODBT when the H_2_O_2_ is present. The incorporated CPPO was used as the chemiluminescent substrate to specifically react with H_2_O_2_, producing the high‐energy intermediate DOD (Figure S1, Supporting Information). Subsequently, through intermolecular electron transfer, the DOD first acquires an electron from the PFODBT, resulting in the carbon dioxide radical anion and the PFODBT radical cation. The cation and anion undergo back electron transfer to create excited PFODBT, which then emits light in the NIR‐I region.^[^ ^15^ ^]^ FRET occurs when the excited PFODBT transfers the received energy to the energy acceptor BPN‐BBTD to generate luminescence in the NIR‐II region.

**Figure 2:**
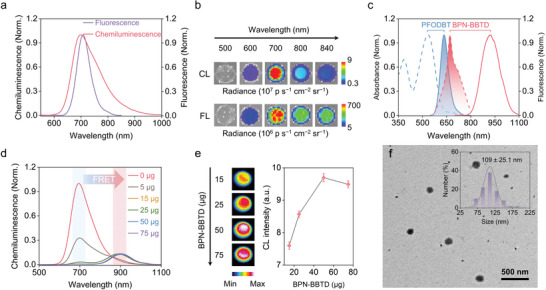
a) Normalized fluorescence emission spectra under the excitation of 350 nm and chemiluminescence emission spectra of PFODBT CL nanoprobes upon the addition of 100 µM H_2_O_2_. b) Chemiluminescence imaging and fluorescence imaging of PFODBT CL nanoprobes in different detection windows (500 ± 10, 600 ± 10, 700 ± 10, 800 ± 10, and 840 ± 10 nm). c) Normalized absorbance and emission spectra of PFODBT and BPN‐BBTD in THF. d) Chemiluminescence spectra of NIR‐II CL nanoprobes with the different mass of BPN‐BBTD upon the addition of 1 mm H_2_O_2_. e) Chemiluminescence images and their corresponding intensity of the different masses of BPN‐BBTD upon the addition of 1 mm H_2_O_2_. f) TEM image of NIR‐II CL nanoprobes. Insert: Size distribution of NIR‐II CL nanoprobes.

To verify the feasibility of the CRET process, only CPPO and PFODBT were embedded into F127 micelles using a nanoprecipitation method (termed PFODBT CL nanoprobes). The synthesized nanoprobes in water exhibited a clear and transparent purple‐red solution (Figure S2, Supporting Information), indicating that PFODBT and CPPO were successfully embedded in the hydrophobic interior of F127. As shown in the transmission electron microscopy (TEM) image, the PFODBT CL nanoprobes were spherical, with a size of 105 ± 28.2 nm (Figure S3, Supporting Information). Compared with PFODBT dissolved in THF, the aqueous solution of PFODBT CL nanoprobes showed a slight red‐shift of the absorption spectra and a significant red‐shift of the fluorescence peak from 621 to 703 nm owing to the J aggregation of the polymer chain (Figure S4, Supporting Information).^[^ ^16^ ^]^ Upon the addition of H_2_O_2_, the PFODBT CL nanoprobes emitted an apparent chemiluminescence signal with an emission peak at 700 nm, which was similar to its fluorescence peak in aqueous solutions (**Figure** **2**a,b, Supporting Information). These results indicated the CRET process successfully occurred in the prepared PFODBT CL nanoprobes. Because the ratio of encapsulated CPPO and PFODBT has a significant impact on CRET efficiency, we optimized the conditions by tuning PFODBT quantities. As the amount of PFODBT used increasing, the absorption peak of PFODBT at 390 and 554 nm gradually increased (Figure S5a, Supporting Information), indicating an enhanced doping concentration of PFODBT in the nanoprobes. However, the chemiluminescence emission of PFODBT progressively grew and plateaued at the amount of 100 µg (Figure S5b,c, Supporting Information). The reduced chemiluminescence signal with higher PFODBT doping may be attributed to small aggregates formation according to aggregation‐caused quenching side effects.^[^ ^17^ ^]^ Therefore, the optimal amount of PFODBT used in the synthesis of the PFODBT CL nanoprobes was determined to be 100 µg, which ensures sufficient loading of PFODBT while avoiding the adverse effects of chemiluminescence quenching induced by aggregation.

For further verifying the feasibility of the FRET process, CPPO, PFODBT, and BPN‐BBTD were coprecipitated into F127 micelles (termed NIR‐II CL nanoprobes). The absorption and fluorescence emission maxima of PFODBT and BPN‐BBTD in THF were 536/635 and 670/925 nm, respectively (Figure 2c). This large Stokes shift ensured that the emission from PFODBT largely overlapped with the absorption signal from BPN‐BBTD, showing the potential feasibility of the FRET process from PFODBT to BPN‐BBTD. With increasing BPN‐BBTD amount from 0 to 75 µg, the chemiluminescence intensity at the peak of 700 nm decreased rapidly, while the peak at 902 nm increased (Figure 2d), confirming the efficient FRET process from PFODBT to BPN‐BBTD. The NIR‐II CL nanoprobe exhibited a chemiluminescence emission at 902 nm, consistent with their corresponding fluorescence spectra (Figure S6, Supporting Information), implying the successful preparation of NIR‐II CL nanoprobes combining CRET and FRET processes to emit NIR‐II luminescence. The strongest NIR‐II emission and an extremely high FRET efficiency (99.48%) were obtained when the BPN‐BBTD amount was 50 µg (Figure 2e and Table S1, Supporting Information). Therefore, the ideal concentration of BPN‐BBTD was found to be 50 µg in subsequent experiments. Under the optimal conditions, the NIR‐II CL nanoprobes showed an average diameter of 109 ± 25.1 nm (TEM, Figure 2f) and a hydrodynamic size of 110.4 nm (Dynamic light scattering, DLS, Figure S7, Supporting Information), respectively. The difference in diameters may be attributed to the different states of the prepared samples. For TEM characterization, the sample was dried on a carbon‐supported copper grid, whereas for DLS, the sample was dispersed in an aqueous solution exhibiting a more “swollen” state.^[^ ^18^ ^]^ To investigate the storage stability of the NIR‐II CL nanoprobes, a potentiometric analysis of the nanoprobes in an aqueous solution was performed for several days. A negligible zeta potential shift was observed for NIR‐II CL nanoprobes during storage for seven days (Figure S8, Supporting Information), demonstrating the excellent colloidal stability of NIR‐II CL nanoprobes. The chemiluminescence intensity of the NIR‐II CL nanoprobes synthesized in six batches showed a similar response signal with a relative standard deviation of 4.2% (Figure S9, Supporting Information), indicating the superior reproducibility of the nanoprobes. In addition, owing to the outstanding structural stability of PFODBT and BPN‐BBTD, micelles containing only two fluorescent molecules without CPPO (termed NIR‐II micelles) exhibited tolerant antioxidant stability even at high concentrations of H_2_O_2_ (Figure S10, Supporting Information).

**Figure 3:**
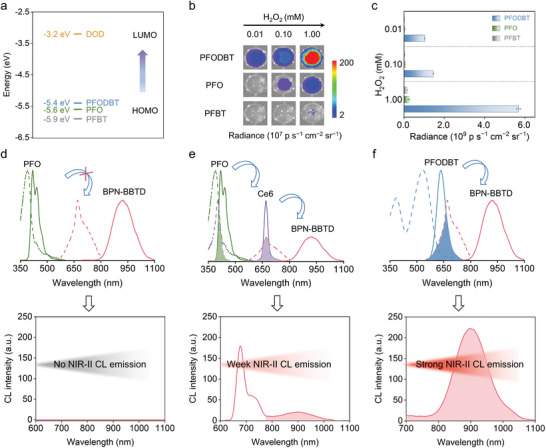
a) Energy levels of PFODBT, PFO, PFBT, and the active DOD intermediate. b) Chemiluminescence images and c) their corresponding chemiluminescence intensity of the PFODBT, PFO, and PFBT CL nanoprobes under different H_2_O_2_ concentrations. Chemiluminescence schemes and NIR‐II chemiluminescence emission spectra of d) not‐matched FRET in PFO and BPN‐BBTD, e) poor‐matched multi‐step FRET in PFO, Ce6, and BPN‐BBTD, and f) well‐matched FRET in PFODBT and BPN‐BBTD.

To achieve the effective chemiluminescent emission in the NIR‐II window, appropriate molecules with good energy‐level matching and substantial Stokes shifts for both energy‐transfer processes (CRET and FRET) are essential. The energy difference between the lowest unoccupied molecular orbital level (LUMO) of the DOD and the highest occupied molecular orbital level (HOMO) of the acceptors must be small to build an effective CRET. To improve this, three semiconducting polymers with different HOMO levels, PFODBT, poly(9,9′‐dioctylfluorenyl‐2,7‐diyl) (PFO), and poly[(9,9′‐dioctylfluorenyl‐2,7‐diyl)‐alt‐(benzo[2,1,3]thiadiazol‐4,7‐diyl)] (PFBT), were combined with CPPO and F127 to synthesize the corresponding nanoprobes and compare their chemiluminescence intensities. The DOD has a LUMO level of −3.2 eV,^[^ ^19^ ^]^ while PFODBT, PFO, and PFBT have HOMO levels of −5.4,^[^ ^20^ ^]^ −5.6,^[^ ^21^ ^]^ and −5.9 eV,^[^ ^22^ ^]^ respectively (**Figure** **3**a and Figure S11, Supporting Information). Compared to the PFO CL nanoprobes and PFBT CL nanoprobes, the PFODBT CL nanoprobes showed the highest chemiluminescence intensity at various concentrations of H_2_O_2_ (Figure 3b,c). This is because the PFODBT HOMO level is the closest to the DOD LUMO level, with the smallest energy gap of 2.2 eV compared to PFO and PFBT, which contributes to the highest chemiluminescence emission. The emission wavelength can be extended to the NIR‐II window by the introduction of effective and well‐matched FRET. The FRET efficiency is dependent on the extent of overlap between the absorption spectrum of the acceptor and the emission spectrum of the donor. To demonstrate this, we embedded CPPO, PFO, and BPN‐BBTD in F127 to construct PFO‐BPN‐BBTD CL nanoprobes and evaluate chemiluminescence emission in the NIR‐II region. The absorption/emission peaks of PFO and BPN‐BBTD were located at 391/420 nm and 670/925 nm, respectively, resulting in negligible overlap between the absorption spectrum of BPN‐BBTD and the emission spectrum of PFO. Therefore, the PFO‐BPN‐BBTD CL nanoprobes failed to generate NIR‐II chemiluminescence (Figure 3d). Furthermore, we designed two‐step FRET nanoprobes by incorporating CPPO, chlorin e6 (Ce6), PFO, and BPN‐BBTD into the F127 micelles (termed PFO‐Ce6‐BPN‐BBTD CL nanoprobes). The absorption and emission spectra of Ce6 (407/670 nm) overlap with the emission spectra of PFO and the absorption spectra of BPN‐BBTD, respectively, resulting in a two‐step FRET. The NIR‐II chemiluminescence signal was identified but was extremely weak owing to the cumulative energy loss in the multi‐step FRET process (Figure 3e). In contrast, in the presence of H_2_O_2_, the NIR‐II CL nanoprobe displayed a strong NIR‐II chemiluminescence emission thanks to the excellent energy match and substantial Stokes shift of PFODBT and BPN‐BBTD (Figure 3f).

**Figure 4:**
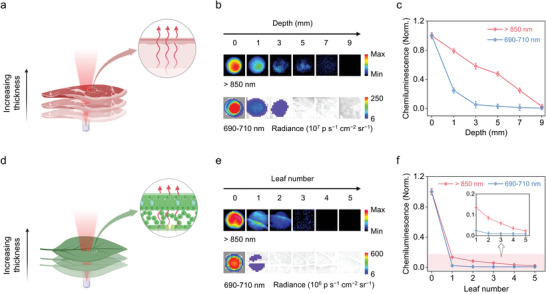
a) Schematic illustration of the light path in chemiluminescence imaging in chicken breasts. b) Chemiluminescence images of NIR‐II CL nanoprobes and PFODBT CL nanoprobes after the addition of 1 mMm H_2_O_2_ through stacked layers of chicken with variable depths in various detection windows (>850 nm and 690–710 nm). c) Corresponding normalized chemiluminescence intensity of NIR‐II CL nanoprobes and PFODBT CL nanoprobes with stacked slices of chicken in (b). d) Schematic illustration of the light path in chemiluminescence imaging in pepper leaves. e) Chemiluminescence images of NIR‐II CL nanoprobes and PFODBT CL nanoprobes through stacked pepper leaves in different detection windows (>850 nm and 690–710 nm). f) Corresponding normalized chemiluminescence intensity of NIR‐II CL nanoprobes and PFODBT CL nanoprobes with stacked pepper leaves in (e).

The tissue penetration depth of chemiluminescence emission in two regions (690–710 nm and >850 nm) was compared to assess the benefit of the chemiluminescence signal in the NIR‐II window. Chicken breast and pepper leaves were used to mimic muscle and plant tissues, respectively (**Figure** **4**a,d). For both detection windows, the chemiluminescence signals in chicken decreased in intensity as tissue depth increased from 0 to 9 mm. The NIR‐II region showed a deeper penetration depth (7 mm) than the visible region (3 mm; Figure 4b,c). The chemiluminescence signal decreased more significantly in the pepper leaves than in the chicken breasts. This may be attributed to the strong absorption (70–90%) of red light (600–700 nm)^[^ ^23^ ^]^ on chlorophyll in green plant tissue.^[^ ^24^ ^]^ However, the NIR‐II region showed deeper penetration depth (three leaf layers) than the visible region (one leaf layer) (Figure 4e,f). Compared with the reported chemiluminescence probes, our NIR‐II CL nanoprobes showed a better or considerable tissue penetration depth (Table S2, Supporting Information), which is appropriate for in vivo deep tissue imaging.

**Figure 5:**
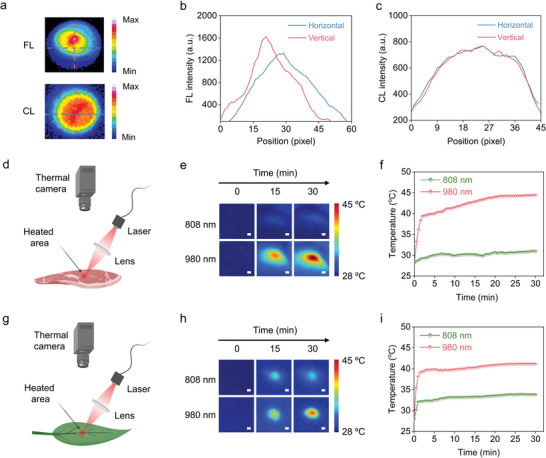
a) Fluorescence and chemiluminescence imaging of NIR‐II CL nanoprobes. b) Fluorescence and c) chemiluminescence intensity profiles in (a). Schematic illustration for the investigation of the photothermal effects on d) chicken and g) leaves. Photothermal imaging of e) chicken and h) peppleaves under constant 808 and 980 nm illumination for 30 min. Scale bar, 5 mm. Temperature fluctuation curves of f) chicken and i) leaves were collected with irradiation of various excitation wavelengths.

Because an external excitation light is not required, chemiluminescence signals avoid the autofluorescence background, overheating effect, and photobleaching from the excitation light that commonly occurs in fluorescence imaging. We compared the luminescent intensity distribution of the NIR‐II CL nanoprobes in a 96‐well plate for fluorescence and chemiluminescence imaging (**Figure** **5**a). In NIR‐II fluorescence imaging, an inhomogeneous signal distribution with an asymmetric intensity profile along the horizontal and vertical directions is observed owing to the excitation light's uneven illumination (Figure 5b).^[^ ^25^ ^]^ In contrast, a uniform signal distribution pattern was seen in NIR‐II chemiluminescence imaging (Figure 5c). Without the need for external excitation, our probe relies on chemical excitation to provide energy, so it does not suffer from unfavorable and inevitably distorted signals in fluorescence imaging. Additionally, the excitation light's tendency to overheat might be detrimental to in vivo long‐term imaging.^[^ ^26^ ^]^ Two laser light sources, 808 and 980 nm, were used to investigate the photothermal effect on chicken breasts (Figure 5d) and pepper leaves (Figure 5g) to mimic muscle and plant tissues. The temperature of the muscle and plant tissue both quickly increased and exceeded 39 °C within 3 min due to the significant water absorption peak at 980 nm,^[^ ^27^ ^]^ which might be harmful to the living body.^[^ ^28^ ^]^ The laser treatment at 808 nm similarly produced a photothermal impact on muscle and plant tissues, resulting in a maximum temperature increase of ≈3 and ≈6 °C (Figure 5e–i), respectively. The photostability of the nanoprobes was assessed by monitoring the fluorescence intensity variation of NIR‐II CL nanoprobes under continuous irradiation with 635 and 808 nm lasers (Figure S12, Supporting Information). After 90 min of continuous excitation, the fluorescence intensity of the NIR‐II CL nanoprobes dropped dramatically, indicating that long‐term laser irradiation has caused significant photobleaching of PFODBT and BPN‐BBTD in the nanoprobes. Thus, these findings suggested that NIR‐II chemiluminescence without external light is a superior option for in vivo imaging.

**Figure 6:**
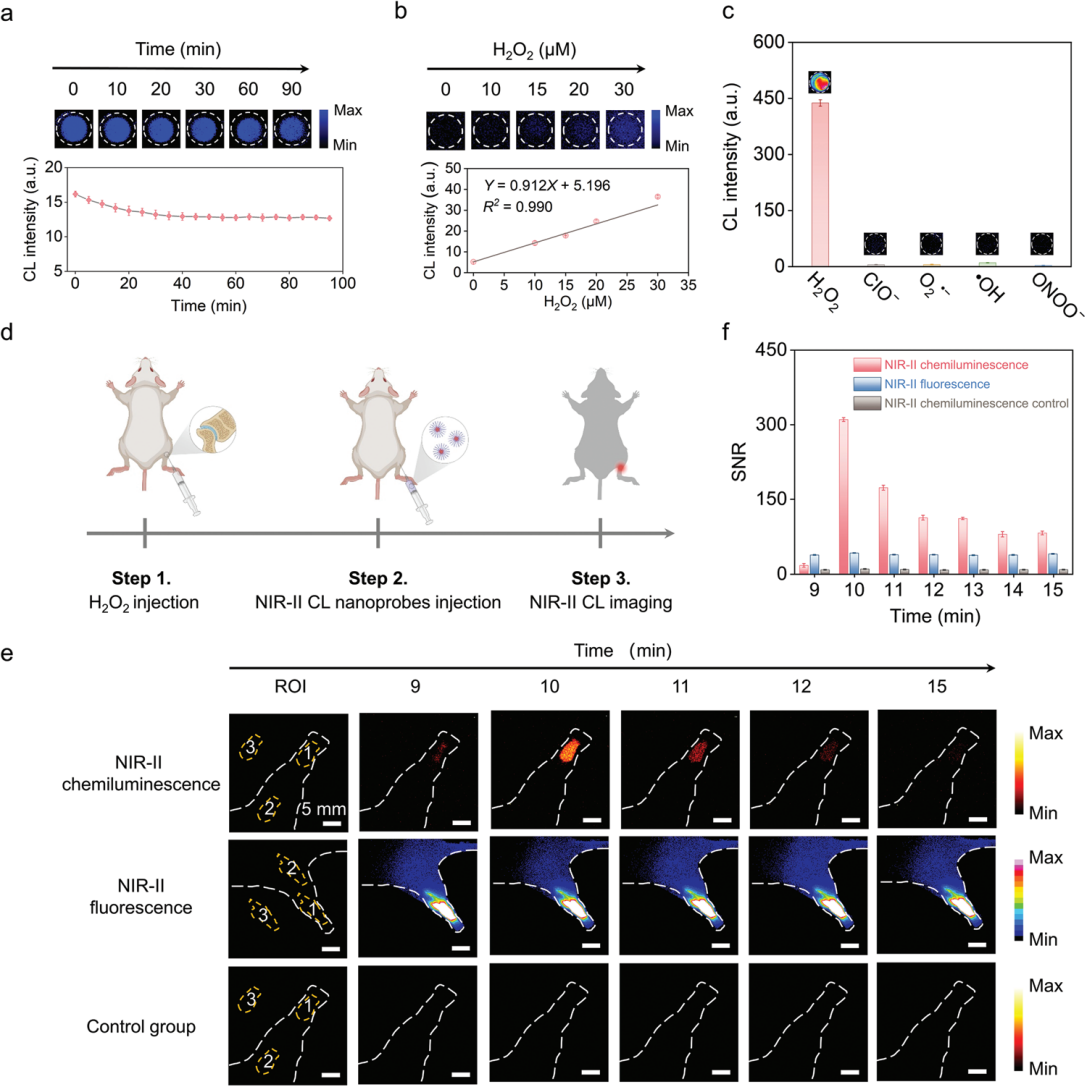
a) Time‐dependent NIR‐II chemiluminescence emission intensities with 100 µm H_2_O_2_. b) Chemiluminescence intensity of NIR‐II CL nanoprobes with different concentrations of H_2_O_2_. c) Chemiluminescence intensity of NIR‐II CL nanoprobes response to various ROS with a concentration of 10 mm. The insert: Corresponding chemiluminescence images after adding the specific ROS. d) Illustration of arthrosis inflammation model of mouse for chemiluminescence imaging. e) Time‐dependent in vivo chemiluminescence imaging of the arthrosis inflammation with NIR‐II CL nanoprobes and corresponding fluorescence imaging. f) Corresponding NIR‐II chemiluminescence and fluorescence SNR as a function of time in e). SNR was defined as SNR = (*I*_ROI1_ – *I*_ROI3_)/(*I*_ROI2_ – *I*_ROI3_).

We further explored the impact of response time and H_2_O_2_ concentration on the effectiveness of chemiluminescence emission. Upon addition of H_2_O_2_ to the NIR‐II CL nanoprobe solution, the chemiluminescence intensity instantaneously reached its maximum value within 0.04 s (Figure S13), indicating the rapid response of the NIR‐II CL nanoprobes to H_2_O_2_. After 30 min, the intensity was still 75% of the maximum value and remained stable for a subsequent time (**Figure** **6**a and Figure S14, Supporting Information). The long‐lasting NIR‐II chemiluminescence signal is attributed to the protective role of the F127 shell for the embedded CPPO, which enables CPPO to gradually react with H_2_O_2_ to release energy to excite neighboring molecules. The long‐lasting NIR‐II chemiluminescence signal of the nanoprobes was suitable for long‐term bioimaging and monitoring in vivo. To further assess the sensitivity of the NIR‐II CL nanoprobe for the H_2_O_2_ response, we measured the chemiluminescence intensity with various concentrations of H_2_O_2_. With increasing H_2_O_2_ concentration, NIR‐II chemiluminescence signals gradually increased (Figure 6b). An excellent linear relationship between the chemiluminescence intensity and H_2_O_2_ concentrations in the range of 0–30 µM was observed. The detection limit (3*σ*/S), where *σ* is the standard deviation of the background noise and S is the slope of the calibration curve, was calculated to be 44.4 nm. The NIR‐II CL nanoprobes have a better or comparable detection limit than the NIR nanoprobes that have previously been reported (Table S3, Supporting Information). Furthermore, it is lower than the physiological levels of H_2_O_2_ concentration in vivo (100 nm),^[^ ^29^ ^]^ implying that our probe has the potential to monitor H_2_O_2_ fluctuations in biological tissues. The selectivity of the NIR‐II CL nanoprobes for the H_2_O_2_ response was evaluated using various reactive oxygen species (ROS) (ClO^−^, O_2_^•−^, ONOO^−^, and •OH) as the interferents. Compared with H_2_O_2_, these ROS triggered negligible responses (Figure 6c), indicating good selectivity of the NIR‐II CL nanoprobe for H_2_O_2_ detection. Human embryonic kidney (HEK293) cells were used to test the NIR‐II CL nanoprobes' cell cytotoxicity utilizing the Cell Counting Kit‐8 (CCK‐8) technique. Cell viability remained above 85% after 24 h of incubation with different concentrations of nanoprobes (Figure S15, Supporting Information), showing satisfactory biocompatibility. Owing to the merits of long‐persistent chemiluminescence emission, high sensitivity, excellent selectivity, and satisfied cytocompatibility, the NIR‐II CL nanoprobe can be used as a powerful molecular probe for H_2_O_2_ imaging in vivo.

To demonstrate the feasibility of NIR‐II CL nanoprobes for in vivo H_2_O_2_ imaging, a detection model of the NIR‐II CL nanoprobes' response to H_2_O_2_ was established by implantation of the mixture of nanoprobes and H_2_O_2_ into the mouse (Figure S16, Supporting Information). The chemiluminescence signal was positively correlated with H_2_O_2_ concentrations, and a linear association was established in the mice for the tested H_2_O_2_ concentrations ranging from 0 to 1 mm (Figure S17, Supporting Information), demonstrating the possibility of in vivo quantitative H_2_O_2_ imaging. Furthermore, a simulated arthrosis inflammation model was developed by injection of H_2_O_2_ and NIR‐II CL nanoprobes in succession (Figure 6d) to investigate the superiority of NIR‐II CL nanoprobes for in vivo imaging. In NIR‐II chemiluminescence imaging, the NIR‐II chemiluminescence signal in the arthrosis inflammation area increased and then decreased owing to the fast flow of H_2_O_2_ in mouse, which implies that our probe can successfully detect in vivo inflammation (Figure 6e). Furthermore, the maximum SNR of the NIR‐II chemiluminescence was as high as 310 after 10 min of NIR‐II CL nanoprobe injection (Figure 6f). Compared with the reported chemiluminescence probes, our NIR‐II CL nanoprobes showed a higher SNR (Table S2, Supporting Information). The control experiment of NIR‐II chemiluminescence exhibited no chemiluminescence signal owing to the lack of H_2_O_2_. The SNR of NIR‐II fluorescence imaging was much lower than that of NIR‐II chemiluminescence, with a maximum SNR of only 42 (Figure 6f), due to the strong tissue background noise brought by external excitation light. Furthermore, we compared the effects of using different long‐pass filters on NIR‐II fluorescence imaging (Figure S18, Supporting Information). Although the tissue background noise decreased as the wavelength of the long‐pass filter increased, the signal of the probe also decreased, resulting in a maximum SNR of only 67, which is still far below that of NIR‐II chemiluminescence. Owing to the extremely low tissue background noise without excitation light, NIR‐II CL nanoprobes can achieve a much better SNR for in vivo inflammatory imaging.

## 3 Conclusion

We designed and synthesized novel NIR‐II CL nanoprobes with efficient NIR‐II chemiluminescence emission. A cascade CRET‐FRET process was applied by choosing suitable dyes with an effective energy level match and a substantial Stokes shift to efficiently extend the chemiluminescence emission to the NIR‐II region. Furthermore, without external light excitation, our self‐luminescent NIR‐II CL nanoprobes eliminated the side effects of signal distortion and tissue overheating risk when performing long‐term imaging compared with fluorescence imaging. In addition, it also has much deeper tissue penetration than traditional chemiluminescence probes with visible or NIR‐I emission. The NIR‐II CL nanoprobes showed excellent analysis performance for the in vitro detection of H_2_O_2_, including good selectivity, long‐lasting luminescence, and high sensitivity with a detection limit of 44.4 nm. Moreover, compared to the NIR‐II fluorescence, NIR‐II CL nanoprobes enable tracking H_2_O_2_ fluctuation in mice with a 7.4‐fold higher SNR. More in vivo sensing and imaging applications may be inspired by NIR‐II chemiluminescence since it is resistant to background interference and light deterioration caused by external excitation light.

## 4 Experimental Section

### Materials

PFODBT (M_w_ > 20 000 g mol^−1^, PFO (M_w_ = 50 000–150 000 g mol^−1^), F127, tetrahydrofuran (THF), sodium hypochlorite (NaOCl), dimethyl sulfoxide (DMSO), and potassium superoxide (KO_2_) were purchased from Sigma–Aldrich. PFBT (M_w_ > 50 000) was purchased from Derthon Optoelectronic Materials Science Technology. CPPO and Ce6 were purchased from the Tokyo Chemical Industry. Hydrogen peroxide (H_2_O_2_, 35 wt.%) and ferrous sulfate heptahydrate (FeSO_4_·7H_2_O) were purchased from Alfa Aesar. Dipotassium hydrogen phosphate trihydrate (K_2_HPO_4_∙3H_2_O) was purchased from Sinopharm. Sodium peroxynitrite (NaONOO) was purchased from Cayman Chemical Co. Dulbecco's Modified Eagle Medium (DMEM) was purchased from HyClone Laboratories. HEK293 cells were obtained from the National Collection of Authenticated Cell Cultures. CCK‐8 was obtained from Beyotime Biotechnology. Fetal Bovine Serum was obtained from Gibco Laboratories. All the materials were used as received without further purification.

### Preparation of Various Micelles

BPN‐BBTD was synthesized according to the literature, with minor modifications.^[^ ^30^ ^]^ The different micelles were prepared using a nanoprecipitation method, and the amounts of ingredients are shown in Table S4 (Supporting Information). In a typical procedure for the NIR‐II CL nanoprobe, different amounts of F127, CPPO, PFODBT, and BPN‐BBTD were dissolved in THF (1 mL) to obtain a stock solution. Under intense sonication for 2 min, the stock solution was quickly injected into the water to produce a clear micellar solution. After THF was removed by heating the micelle solution at 45 °C for 12 h, to get rid of big aggregates, the aqueous solution was filtered through a polyvinylidene fluoride filter (0.22 µm, Millipore, USA). The obtained suspension was finally concentrated to 500 µL through ultrafiltration (Amicon Ultra‐15 30 KD, Millipore, USA) and used immediately for experiments.

### Characterization

TEM characterization was performed using a JEM‐1200EX transmission electron microscope (JEOL, Japan). DLS was performed using a Zetasizer Nano ZS90 instrument (Malvern Instruments, UK). UV–Vis–NIR absorption spectra were collected using an Agilent 8453 UV‐Vis spectrophotometer (G1103A, Agilent Technologies, Germany). The visible and NIR‐I fluorescence spectra were recorded using an F‐7000 fluorescence spectrophotometer (Hitachi, Japan). NIR‐II fluorescence and chemiluminescence spectra were collected using a QE‐Pro spectrometer (Ocean Optics, USA).

### Fluorescence and Chemiluminescence Imaging system

A custom imaging system with a 640 × 512‐pixel 2D InGaAs NIRvana‐LN CCD camera (Princeton Instruments, USA) was used for NIR‐II fluorescence and chemiluminescence imaging. A 635 nm laser (Changchun New Industries Optoelectronics Technology, China) and an 850 nm long‐pass filter (Thorlabs, USA) were employed for NIR‐II fluorescence imaging. The exposure times for NIR‐II chemiluminescence and fluorescence imaging were set to 20 and 1 s, respectively. The obtained images were analyzed using ImageJ software. An IVIS Spectrum imaging system (PerkinElmer, USA) with auto‐acquisition time was used to carry out the visible and NIR‐I fluorescence and chemiluminescence imaging. For fluorescence imaging, a 430 nm laser was used as the excitation light, and a series of band‐pass filters (500 ± 10, 600 ± 10, 700 ± 10, 800 ± 10, or 840 ± 10 nm) was used. Images collected with the IVIS Spectrum imaging system were processed using Living Image 4.0.

### Calculation of FRET Efficiency

The formula E = 1 – *I*/*I*_0_ was used to calculate the FRET efficiency between organic dyes, where *I* and *I*_0_ represent the fluorescence intensities of the donor PFODBT with and without the acceptor BPN‐BBTD, respectively.^[^ ^31^ ^]^ The FRET efficiency of the NIR‐II CL nanoprobes between PFODBT and BPN‐BBTD at different concentrations was calculated according to the equation (Table S1, Supporting Information).

### Illumination Homogeneity Comparison

The luminescence intensity distribution of NIR‐II CL nanoprobes in the NIR‐II chemiluminescence (without excitation) and NIR‐II fluorescence imaging (635 nm excitation) modes was compared for investigating how inhomogeneous lighting influences the imaging results. Ten microliters of 10 mm H_2_O_2_ in 100 mm K_2_HPO_4_ solution or 10 µL of 100 mm K_2_HPO_4_ solution were mixed with 90 µL of NIR‐II CL nanoprobes for NIR‐II chemiluminescence or NIR‐II fluorescence imaging, respectively.

### Photothermal Effects Comparison

Two commonly used excitation lasers (808 and 980 nm) were chosen to study the photothermal effects. All laser beams were collimated and performed with the same output power density (0.5 W cm^−2^) and a diameter of ≈25 mm. The temperature was measured with a thermal imaging Fluke Ti400 infrared camera (Fluke Corporation, USA). The maximum temperature was calculated from the thermal images using Fluke software.

### H_2_O_2_ Detection Performance In Vitro

In a typical process for H_2_O_2_ detection in vitro, 90 µL of NIR‐II CL nanoprobes was mixed with 10 µL of H_2_O_2_ in a 100 mm K_2_HPO_4_ solution at different concentrations. The mixture was then put into a black 96‐well plate for NIR‐II chemiluminescence imaging. Chemiluminescence intensity was calculated from a region of interest in the images.

### Preparation of Various Reactive Oxygen Species

Commercially available H_2_O_2_, NaONOO, and NaClO were directly diluted to prepare H_2_O_2_, ONOO^−^, and ClO^−^ stock solutions. Hydroxy radical (•OH) was produced via Fenton chemistry by the addition of Fe^2+^ solution into excess H_2_O_2_. O_2_^•−^ was produced by the addition of solid KO_2_ in the aqueous solution. Subsequently, 10 µL of the as‐prepared ROS stock solution was mixed with 90 µL of NIR‐II CL nanoprobes to achieve a final concentration of 10 mM for chemiluminescence measurement immediately.

### Cytotoxicity Test

HEK293 cells were used to test the NIR‐II CL nanoprobes' cell cytotoxicity using CCK‐8 technique. Cells were grown in DMEM with 10% fetal bovi+ne serum in a humidified incubator with 5% CO_2_ at 37 °C. After the cells had been seeded in a 96‐well plate for 24 h, the NIR‐II CL nanoprobes were added and incubated with the cells for another 24 h. Ten microliters of CCK‐8 was added to each well and incubated at 37 °C for 4 h. The absorbance of CCK‐8 at 450 nm was recorded on a microplate reader (EPOCH 2, BioTek Instrument). The formula, viability = (absorbance of cells and nanoprobes/absorbance of cells and culture medium only) × 100%, was used to determine the cell viability.

### Kinetic Study

The reaction kinetics of the nanoprobes for H_2_O_2_ was measured by automatically mixing nanoprobes (10 µL) with H_2_O_2_ (50 mm, 10 µL) in the dual‐channel mixing chamber on a stopped‐flow spectrometer (SX 20, Applied Photophysics, UK). The chemiluminescence intensity of the nanoprobes was recorded over time at a test voltage of 790 V.

### Animal Models

All animal procedures complied with the guidelines of the Institutional Ethical Committee of Animal Experimentation at Zhejiang University (ZJU20210222). Male mice that were 5 weeks old were bought from Shanghai SLAC Laboratory Animal Co. Ltd. Three mice were independently investigated in each group of the imaging experiments. Before imaging, all mice received a pentobarbital injection to make anesthesia.

### In Vivo Chemiluminescence Imaging

Ninety microliters of NIR‐II CL nanoprobes were reacted with 10 µL of H_2_O_2_ at 0.1, 0.5, and 1 mm. The mixture was immediately injected subcutaneously into the mice. Chemiluminescence images were acquired with a 20 s acquisition time using a 640 × 512‐pixel 2D InGaAs NIRvana‐LN CCD camera.

### In Vivo Chemiluminescence Imaging of Arthrosis Inflammation

H_2_O_2_ (10 mm, 10 µL) was injected intra‐articularly to create the arthrosis inflammation model. After 1 min, 75 µL of NIR‐II CL nanoprobes was injected into the arthrosis of the mouse. Afterward, the treated mouse were immediately transferred into a home‐built small‐animal imaging system with a 640 × 512‐pixel 2D InGaAs NIRvana‐LN CCD camera for NIR‐II chemiluminescence imaging. The ideal wavelength of the NIR‐II light was chosen using an 850 nm long‐pass filter. The exposure time was set at 20 s. In the control experiments, 75 µL of NIR‐II CL nanoprobes were injected into the arthrosis of healthy mice for direct imaging. The NIR‐II images were analyzed using ImageJ software.

### In Vivo Fluorescence Imaging of Mouse Arthrosis

For fluorescence imaging, 75 µL of NIR‐II CL nanoprobes were injected into the arthrosis of the mouse. Afterward, the treated mice was immediately transferred into a home‐built small‐animal imaging system with a 640 × 512‐pixel 2D InGaAs NIRvana‐LN CCD camera for NIR‐II fluorescence imaging. An 808 nm laser was used as the excitation light. Long‐pass filters of 850, 900, and 1000 nm were applied throughout the animal imaging experiments, and the exposure time was 800 ms. The NIR‐II images were analyzed using ImageJ software.

## Conflict of Interest

The authors declare no conflict of interest.

## Supporting information

Supporting Information

## Data Availability

The data that support the findings of this study are available from the corresponding author upon reasonable request.
