# Supplementary material for: Self‐Illuminating NIR‐II Chemiluminescence Nanosensor for In Vivo Tracking H2O2 Fluctuation
Source: Adv Sci (Weinh). 2023 Jun 13;10(23):2207651. doi: 10.1002/advs.202207651 (PMC10427367; doi:10.1002/advs.202207651)
Supplement: Supplementary file 1 — Supporting Information [file ADVS-10-2207651-s001.pdf]

ADVANCED SCIENCE
Open Access

Supporting Information

for *Adv. Sci.*, DOI 10.1002/advs.202207651

Self-Illuminating NIR-II Chemiluminescence Nanosensor for In Vivo Tracking H_2O_2 Fluctuation

*Shiyi Zhang, Hao Yuan, Shengchun Sun, Chunlian Qin, Qiming Qiu, Yuyan Feng, Yongjie Liu, Yang Li, Lizhou Xu, Yibin Ying, Ji Qi and Yixian Wang**

Self-Illuminating NIR-II Chemiluminescence Nanosensor for *In Vivo* Tracking H_2O_2 Fluctuation

Shiyi Zhang, Hao Yuan, Shengchun Sun, Chunlian Qin, Qiming Qiu, Yuyan Feng, Yongjie Liu, Yang Li, Lizhou Xu, Yibin Ying, Ji Qi, Yixian Wang[*]

S. Y. Zhang, S. C. Sun, H. Yuan, Dr. Q. M. Qiu, Y. Y. Feng, Prof. Y. B. Ying, Prof. Y. X. Wang

School of Biosystems Engineering and Food Science, Zhejiang University, Hangzhou 310058, China

Key Laboratory of Intelligent Equipment and Robotics for Agriculture of Zhejiang Province, Hangzhou 310058, China

C. L. Qin, Prof. L. Z. Xu, Prof. Y. B. Ying, Prof. Y. X. Wang

ZJU-Hangzhou Global Scientific and Technological Innovation Center, Hangzhou 311215, China

E-mail: yixianwang@zju.edu.cn

Y. J. Liu, Y. Li

Children's Hospital, Zhejiang University School of Medicine, National Clinical Research Center for Child Health, National Children's Regional Medical Center, Hangzhou 310052, China

Prof. J. Qi

Frontiers Science Center for Cell Responses, State Key Laboratory of Medicinal Chemical Biology, Key Laboratory of Bioactive Materials, Ministry of Education, and College of Life Sciences, Nankai University, Tianjin 300071, China

Table S1. FRET efficiency of NIR-II CL nanoprobes with PFODBT and different amounts of BPN-BBTD.

BPN-BBTD (μg)	0	5	15	25	50	75
**Chemiluminescence (700 nm)**	1650.04	544.47	86.22	51.04	8.58	13.22
**FRET efficiency (%)**	0.00	67.00	94.77	96.91	99.48	99.20

Table S2. Performance comparison of the reported chemiluminescence probes with the NIR-II CL nanoprobes designed by us.

**CL substrate**	**CRET acceptor ($\lambda_{ex}/\lambda_{em}$, nm/nm)**	**FRET acceptor ($\lambda_{ex}/\lambda_{em}$, nm/nm)**	**FRET efficiency (%)**	**Penetration depth (mm)**	**CL duration *in vitro* *(min)***	**Detection window *in vivo* *(nm)***	**SNR *in vivo***	**Ref**
CPPO	BDSA (430/584)	-	-	-	-	-	-	[S1]
CPPO	BLSA (412/607)	Nile Red (-/625)	81	~7	-	Open filter		[S2]
CPPO	Pentacene (-/630)	-	-	>3	-	-	-	[S3]
CPPO	r-CD (330/634)	-	-	-	~3	-	-	[S4]
CPPO	TPETPAFN (497/660)	-	-		>30	-		[S5]
CPPO	TPE-BT-DC (460/660)	-	-	-	360	-	-	[S6]
TCPO	Ce6 (-/670)	-	-	-	-	-	-	[S7]
CPPO	Cy5 (-/675)	-	-	-	60	-	-	[S8]
CPPO	PFODBT (540/695)	-	-	-	3600	-	-	[S9]
TCPO	PFPV (452/507)	NIR775 (773/775)	-	-	~100	780±10	-	[S10]
CPPO	PFODBT (580/680)	IR775S (775/820)	-	-	-	Open filter	-	[S11]
CPPO	BTD540 (540/700)	TPE-BBT (680/950)	-	-	-	>900	130	[S12]
CPPO	BTD540 (540/680)	BBTD700 (700/985)	94.12	~8	~80	>850	5.85	[S13]
CPPO	PFODBT (536/635)	BPN-BBTD (670/925)	99.48	~7	>95	>850	310	This work

Table S3. Sensing performance comparison of the NIR chemiluminescence nanoprobes for H_2O_2 detection.

**Nanoprobe components**	**Emission (nm)**	**Linear range (μM)**	**Detection limit (nM)**	**Ref.**
Peroxalate polymer/Pentacene	630	0-10	250	[S3]
CPPO/Cy5	701	0.01-10	100	[S8]
CPPO/BLSA	556	0-10	100	[S2]
CPPO/PFODBT/IR775S	820	0-8	5	[S11]
TCPO/PFPV/NIR775	775	0.008-10	8	[S10]
CPPO/PFO/Hemin	-	0-500	250	[S9]
CPPO/BTD540/TPE-BBT	910	8-800	-	[S12]
CPPO/BTD540/BBTD700	935	0-22	17400	[S13]
CPPO/PFODBT/BPN-BBTD	902	0-30	44	This work

Table S4. Amount of F127, CPPO, PFODBT, PFO, PFBT, Ce6, and BPN-BBTD in the different nanoprobes.

Name of nanoprobe	F127 (mg)	CPPO (mg)	PFODBT (μg)	PFO (μg)	PFBT (μg)	Ce6 (μg)	BPN-BBTD (μg)
PFODBT CL nanoprobes	10	10	0	-	-	-	-
	10	10	10	-	-	-	-
	10	10	50	-	-	-	-
	10	10	100	-	-	-	-
	10	10	200	-	-	-	-
	10	10	400	-	-	-	-
NIR-II CL nanoprobes	10	10	100	-	-	-	5
	10	10	100	-	-	-	15
	10	10	100	-	-	-	25
	10	10	100	-	-	-	50
	10	10	100	-	-	-	75
NIR-II micelles	10	-	100	-	-	-	50
PFO CL nanoprobes	10	10	-	100	-	-	-
PFBT CL nanoprobes	10	10	-	-	100	-	-
PFO-BPN-BBTD CL nanoprobes	10	10	-	100	-	-	50
PFO-Ce6-BPN-BBTD CL nanoprobes	10	10	-	250	-	100	50

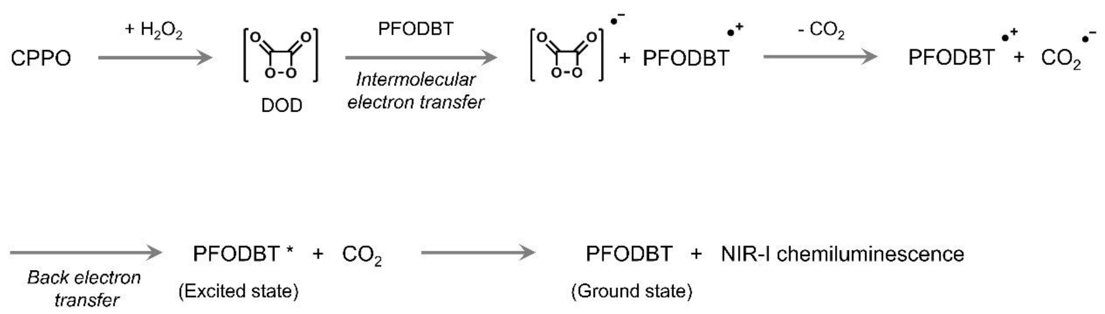

Figure S1. Illustration of the CIEEL mechanism of CRET.

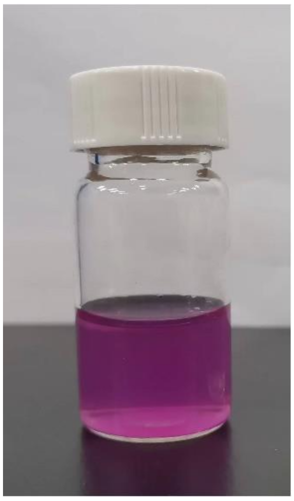

Figure S2. Photograph of PFODBT CL nanoprobes in aqueous solution. The amount of PFODBT, CPPO, and F127 is 100 μg, 10 mg, and 10 mg, respectively.

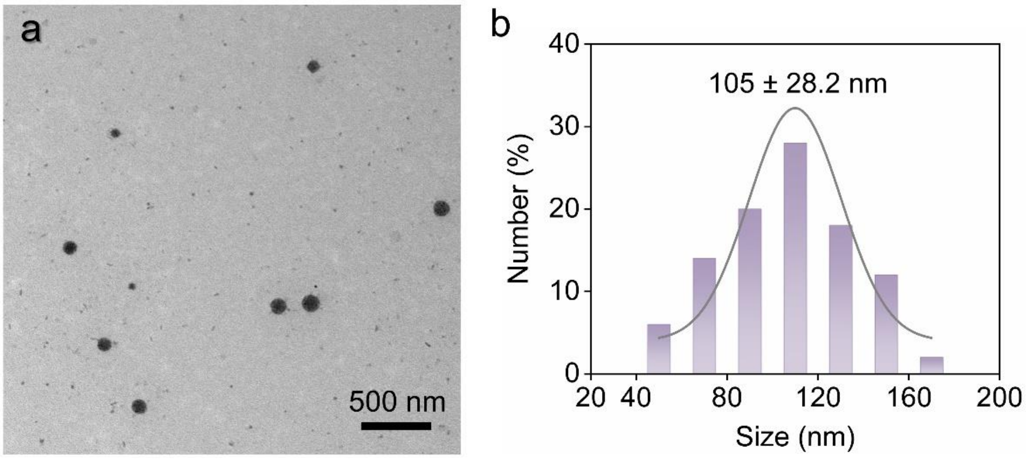

Figure S3. a) TEM image of PFODBT CL nanoprobes. b) Size distribution of 50 randomly selected PFODBT CL nanoprobes.

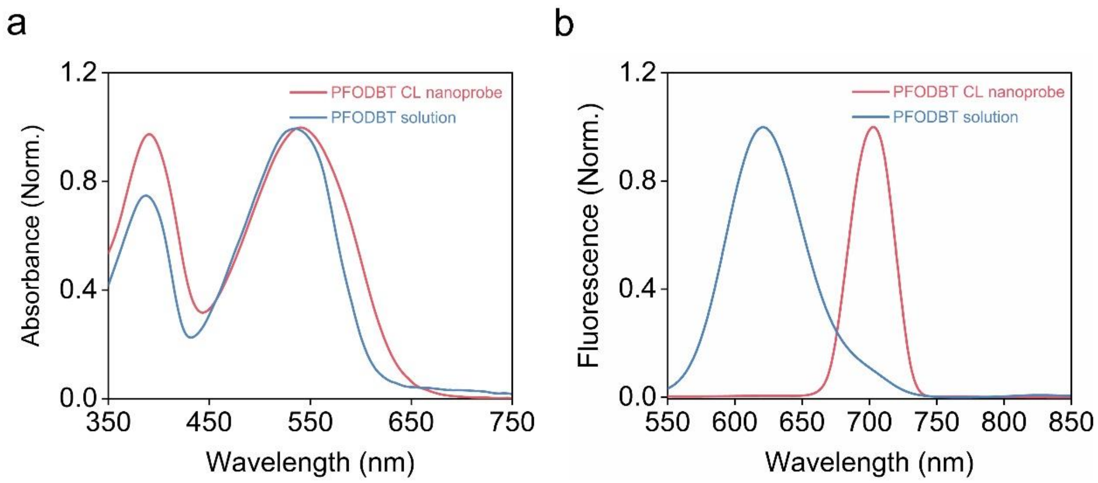

Figure S4. a) Absorption spectrum of PFODBT CL nanoprobes in aqueous solution and PFODBT in THF. b) Fluorescence emission spectrum of PFODBT CL nanoprobes in aqueous solution and PFODBT in THF (λ_{ex} = 350 nm).

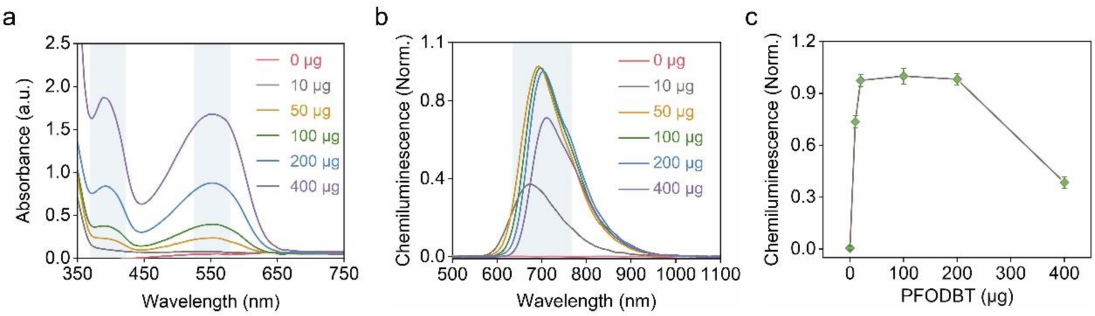

Figure S5. a) Absorption spectra, b) normalized chemiluminescence spectra, and c) corresponding chemiluminescence intensity of PFODBT CL nanoprobes with different amounts of PFODBT. The amount of CPPO and F127 used in the nanoprobes is 10 mg and 10 mg, respectively.

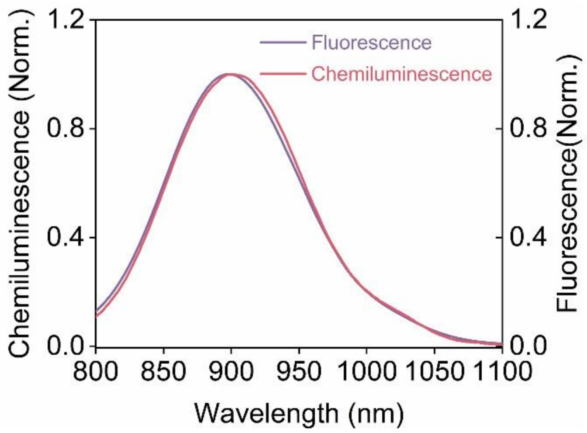

Figure S6. Normalized fluorescence emission spectrum under the excitation of 808 nm and chemiluminescence emission spectrum of NIR-II CL nanoprobes upon the addition of 1 mM H_2O_2.

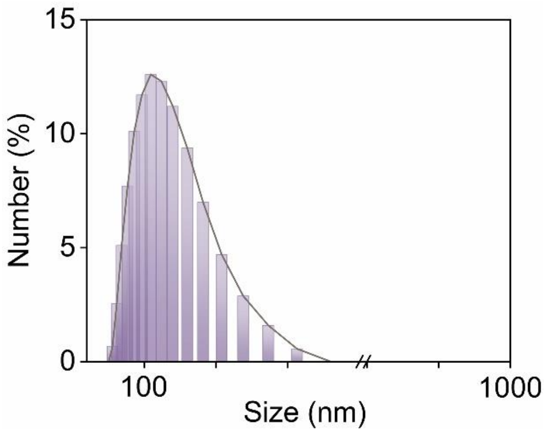

Figure S7. DLS profiles of NIR-II CL nanoprobes.

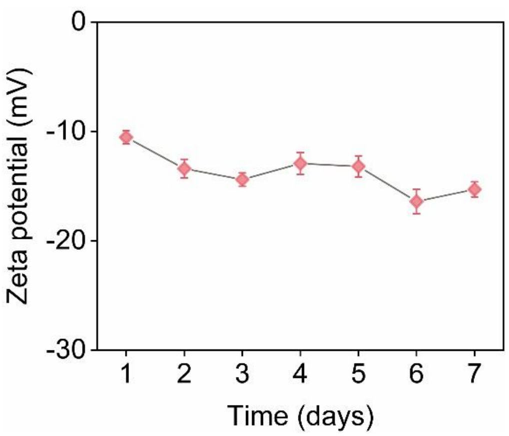

Figure S8. Stability of NIR-II CL nanoprobes under storage at room temperature for seven days.

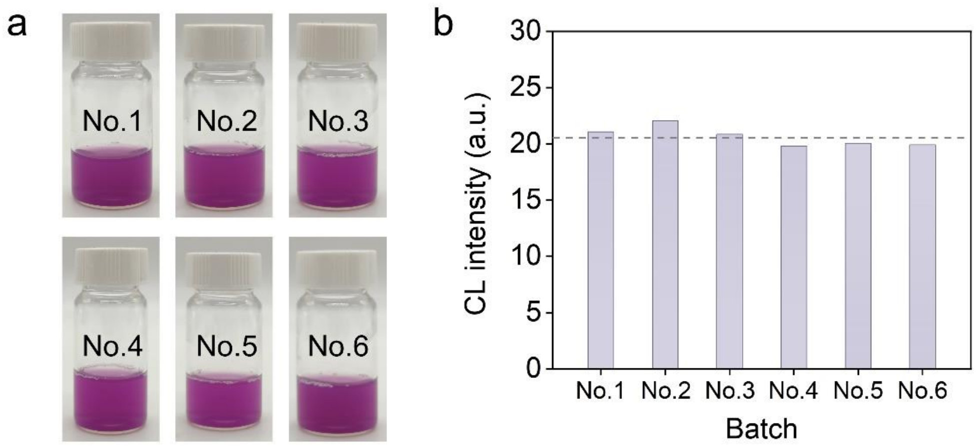

Figure S9. Reproducibility of the NIR-II CL nanoprobes. a) Photograph and b) corresponding chemiluminescence signal of the NIR-II CL nanoprobes for six batches.

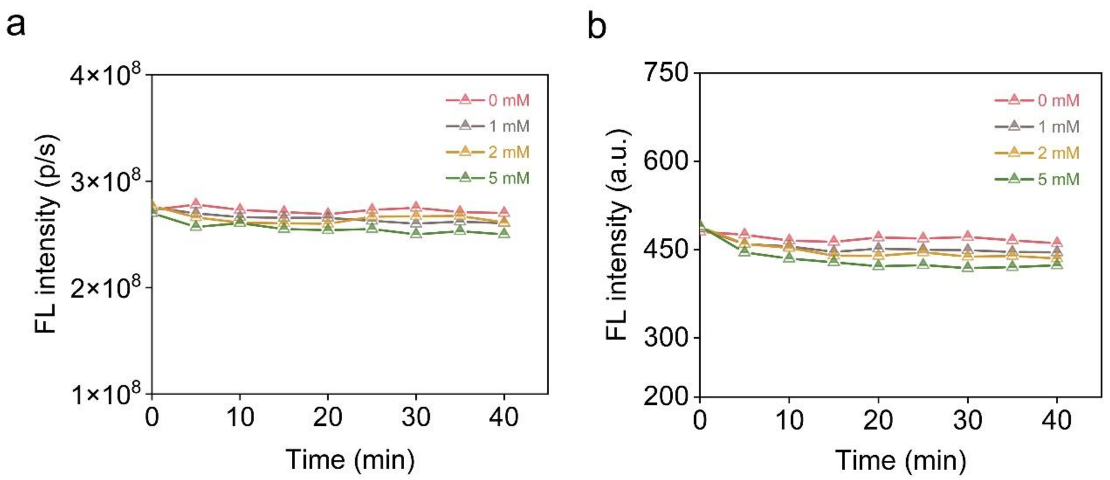

Figure S10. a) Plot of fluorescence emission intensity changes of NIR-II micelles at 690-710 nm as a function of H_2O_2 concentrations (0-5 mM) and time (0-40 min) under 430 nm excitation. b) Plot of fluorescence emission intensity changes of NIR-II micelles under 850 nm long-pass filter as a function of H_2O_2 concentrations (0-5 mM) and time (0-40 min) under 635 nm excitation.

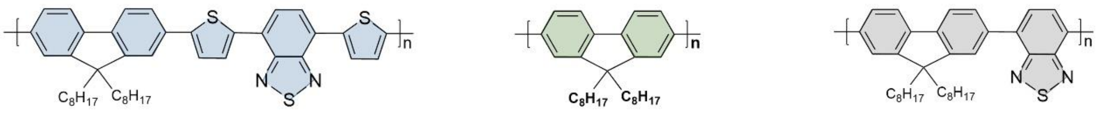

PFODBT PFO PFBT

Figure S11. Chemical structures of PFODBT, PFO, and PFBT.

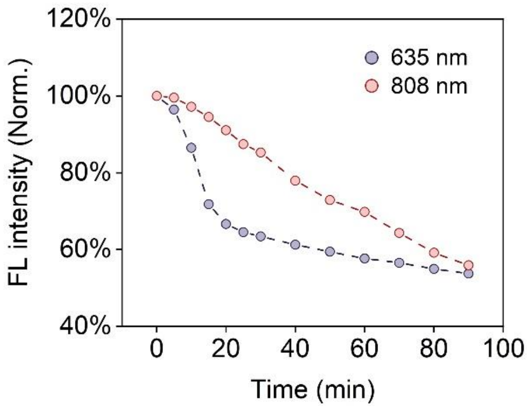

Figure S12. Fluorescence emission intensity at 700 and 902 nm of the NIR-II CL nanoprobes upon continuous irradiation with 635 nm and 808 nm lasers at a power density of 0.5 W cm^{-2}.

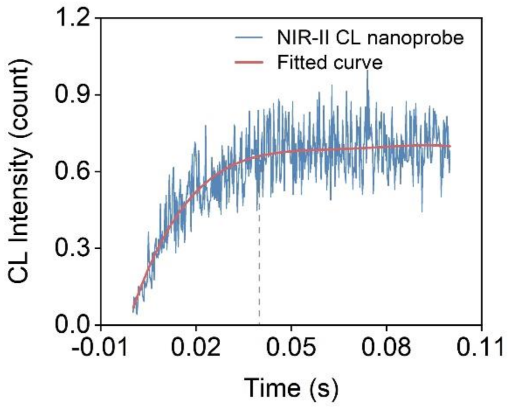

Figure S13. Chemiluminescence kinetic curves of the response of NIR-II CL nanoprobes to H_2O_2.

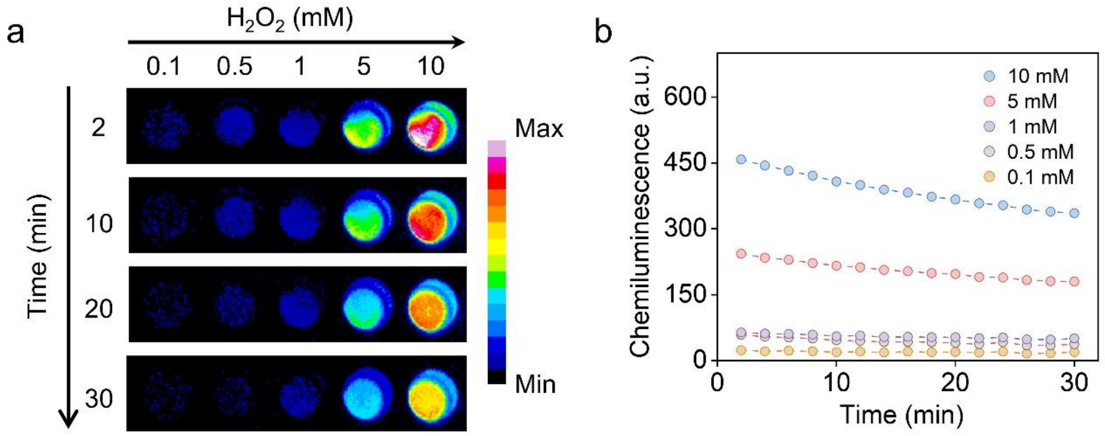

Figure S14. a) Time-dependent chemiluminescence imaging and b) corresponding chemiluminescence signal of NIR-II CL nanoprobes upon addition of different concentrations of H_2O_2.

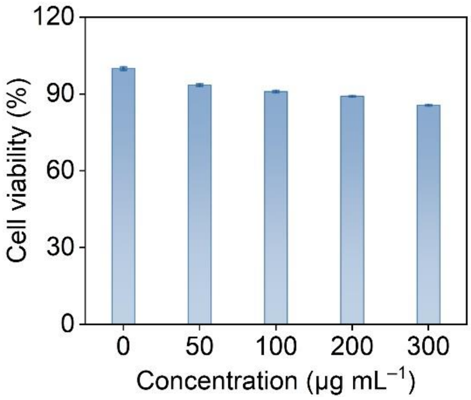

Figure S15. Cytotoxicity studies of NIR-II CL nanoprobes at different concentrations.

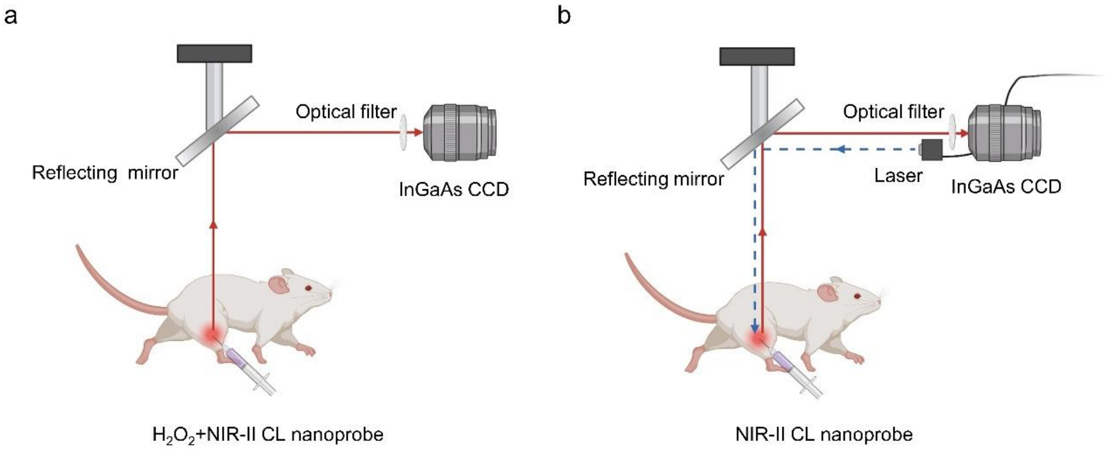

Figure S16. Schemes of a) NIR-II chemiluminescence imaging and b) NIR-II fluorescence imaging.

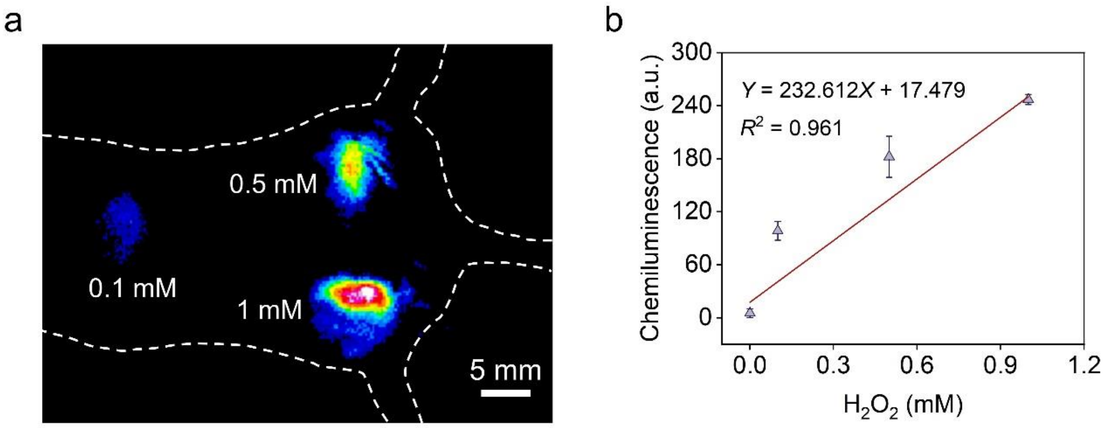

Figure S17. a) Representative chemiluminescence image of the mouse with the subcutaneous implantation of the NIR-II CL nanoprobes with different concentrations of H_2O_2. b) Corresponding chemiluminescence intensity of the subcutaneous inclusion of NIR-II CL nanoprobes as a function of the H_2O_2 concentrations.

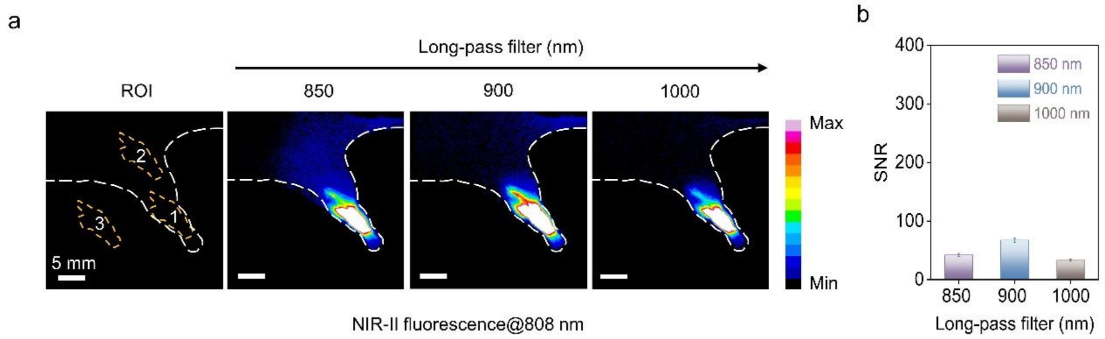

Figure S18. a) *In vivo* fluorescence imaging of NIR-II CL nanoprobes (808 nm excitation) with different long-pass filters and b) corresponding SNR.

References

[S1] A. Singh, Y. H. Seo, C.-K. Lim, J. Koh, W.-D. Jang, I. C. Kwon, S. Kim, *ACS Nano* **2015**, *9*, 9906.

[S2] Y.-D. Lee, C.-K. Lim, A. Singh, J. Koh, J. Kim, I. C. Kwon, S. Kim, *ACS Nano* **2012**, *6*, 6759.

[S3] D. Lee, S. Khaja, J. C. Velasquez-Castano, M. Dasari, C. Sun, J. Petros, W. R. Taylor, N. Murthy, *Nat. Mater.* **2007**, *6*, 765.

[S4] C. L. Shen, Q. Lou, C. F. Lv, J. H. Zang, S. N. Qu, L. Dong, C. X. Shan, *Adv. Sci.* **2019**, *6*, 1802331.

[S5] J. Geng, K. Li, W. Qin, B. Z. Tang, B. Liu, *Part. Part. Syst. Charact.* **2014**, *31*, 1238.

[S6] D. Mao, W. Wu, S. Ji, C. Chen, F. Hu, D. Kong, D. Ding, B. Liu, *Chem* **2017**, *3*, 991.

[S7] J. Jeon, D. G. You, W. Um, J. Lee, C. H. Kim, S. Shin, S. Kwon, J. H. Park, *Sci. Adv.* **2020**, *6*, 8400.

[S8] C. K. Lim, Y. D. Lee, J. Na, J. M. Oh, S. Her, K. Kim, K. Choi, S. Kim, I. C. Kwon, *Adv. Funct. Mater.* **2010**, *20*, 2644.

[S9] Y. Wang, L. Shi, Z. Ye, K. Guan, L. Teng, J. Wu, X. Yin, G. Song, X.-B. Zhang, *Nano Lett.* **2019**, *20*, 176.

[S10] X. Zhen, C. Zhang, C. Xie, Q. Miao, K. L. Lim, K. Pu, *ACS Nano* **2016**, *10*, 6400.

[S11] A. J. Shuhendler, K. Pu, L. Cui, J. P. Uetrecht, J. Rao, *Nat. Biotechnol.* **2014**, *32*,

373.

[S12] H. Shen, F. Sun, X. Zhu, J. Zhang, X. Ou, J. Zhang, C. Xu, H. H. Sung, I. D. Williams, S. Chen, *J. Am. Chem. Soc.* **2022**, *144*, 15391.

[S13] Y. Yang, S. Wang, L. Lu, Q. Zhang, P. Yu, Y. Fan, F. Zhang, *Angew. Chem.* **2020**, *132*, 18538.
